# NAMPT-dependent NAD^+^ biosynthesis controls circadian metabolism in a tissue-specific manner

**DOI:** 10.1073/pnas.2220102120

**Published:** 2023-03-30

**Authors:** Astrid L. Basse, Karen N. Nielsen, Iuliia Karavaeva, Lars R. Ingerslev, Tao Ma, Jesper F. Havelund, Thomas S. Nielsen, Mikkel Frost, Julia Peics, Emilie Dalbram, Morten Dall, Juleen R. Zierath, Romain Barrès, Nils J. Færgeman, Jonas T. Treebak, Zachary Gerhart-Hines

**Affiliations:** ^a^Novo Nordisk Foundation Center for Basic Metabolic Research, Faculty of Health and Medical Sciences, University of Copenhagen, DK-2200 Copenhagen, Denmark; ^b^Department of Biochemistry and Molecular Biology, Villum Center for Bioanalytical Sciences, University of Southern Denmark, DK-5230 Odense, Denmark; ^c^Department of Molecular Medicine and Surgery, Section of Integrative Physiology, Karolinska Institutet, SE-171 77 Stockholm, Sweden; ^d^Institut de Pharmacologie Moléculaire et Cellulaire, Université Côte d'Azur and CNRS, 06560 Valbonne, France

**Keywords:** Circadian metabolism, Clock rhythm, Brown adipose tissue, Skeletal muscle, NAD

## Abstract

Molecular clocks in the periphery coordinate tissue-specific daily biorhythms by integrating input from the hypothalamic master clock and intracellular metabolic signals. One such key metabolic signal is the cellular concentration of NAD^+^, which oscillates along with its biosynthetic enzyme, nicotinamide phosphoribosyltransferase (NAMPT). NAD^+^ levels feed back into the clock to influence rhythmicity of biological functions, yet whether this metabolic fine-tuning occurs ubiquitously across cell types and is a core clock feature is unknown. Here, we show that NAMPT-dependent control over the molecular clock varies substantially between tissues. Brown adipose tissue (BAT) requires NAMPT to sustain the amplitude of the core clock, whereas rhythmicity in white adipose tissue (WAT) is only moderately dependent on NAD^+^ biosynthesis, and the skeletal muscle clock is completely refractory to loss of NAMPT. In BAT and WAT, NAMPT differentially orchestrates oscillation of clock-controlled gene networks and the diurnality of metabolite levels. NAMPT coordinates the rhythmicity of TCA cycle intermediates in BAT, but not in WAT, and loss of NAD^+^ abolishes these oscillations similarly to high-fat diet-induced circadian disruption. Moreover, adipose NAMPT depletion improved the ability of animals to defend body temperature during cold stress but in a time-of-day-independent manner. Thus, our findings reveal that peripheral molecular clocks and metabolic biorhythms are shaped in a highly tissue-specific manner by NAMPT-dependent NAD^+^ synthesis.

## Significance

We evaluate the role of NAD^+^ circadian control by comparing diurnal gene expression across brown adipose tissue (BAT), white adipose tissue (WAT), and skeletal muscle of mice with tissue-specific deletion of the NAD^+^ biosynthetic enzyme nicotinamide phosphoribosyltransferase (NAMPT). Despite a substantial reduction in NAD^+^ levels across all three tissues, the outcomes on the clock are markedly different. We observe profound attenuation of core clock oscillation in response to *Nampt* deletion in BAT, moderate attenuation in WAT, and no changes in skeletal muscle. Furthermore, *Nampt* deletion reduced the number of rhythmic transcripts and metabolites in BAT. Our study underscores the importance of investigating in vitro clock mechanisms across different organs to determine their ubiquitous versus cell-specific contributions to circadian biology in vivo.

Temporal regulation of metabolism has been described in a vast range of species, from archaea to mammals (1, 2). This regulation allows for anticipation of changes in environment and behavior such as light, sleep, and food intake, and ensures entrainment of molecular programs with these changes. In mice, misalignment of behavioral patterns with normal circadian rhythms results in dysregulation of metabolism and obesity (3). In humans, the disruption of normal circadian rhythms has been thoroughly studied in shift workers, who are more likely to gain weight, have high BMI, and exhibit higher waist-to-hip ratios (4–11). Several genetic variants of core clock genes are associated with metabolic disease (12–18). Thus, chronodisruption appears to be a contributing factor of the obesity epidemic and understanding the interaction between weight gain and the clock may unravel paths for targeting metabolic diseases.

The circadian clock in mammals consists of interlocked transcription/translation feedback loops with genes oscillating in periods of about 24 h. The CLOCK:BMAL1 heterodimer acts as a transcription factor that regulates the transcription of period (*Per*) and cryptochrome (*Cry*) genes. Gene products of *Per* and *Cry* block the function of CLOCK:BMAL1 and thereby repress *Per* and *Cry* transcription in a negative feedback fashion. CLOCK:BMAL1 also enhances the transcription of Reverb-α and β genes (*Nr1d1 and Nr1d2*) as well as *Ror* genes, which act directly as transcriptional repressors for *Bmal1*. The oscillating core clock components in turn control the circadian transcription pattern of metabolic genes to entrain various gene networks to a specific time of day and behavior (19).

The clock is also regulated directly by several noncore clock genes, one of which is the NAD^+^-dependent deacetylase sirtuin 1 (SIRT1). SIRT1 deacetylates histones located at promoters of circadian genes (20), and it deacetylates PER2, which promotes its degradation (21), and BMAL1 to relieve CRY1 suppression (20). SIRT1 is therefore a major influencer on the clock. Oscillations in SIRT1 activity is thought, in part, to be driven by oscillating levels of its cofactor NAD^+^ and the rate-limiting enzyme in the NAD^+^ salvage pathway, nicotinamide phosphoribosyltransferase (NAMPT) (22, 23). *Nampt* expression is itself controlled directly by CLOCK:BMAL1 and SIRT1 (23). In adipose tissue, *Nampt* expression is decreased in response to obesity in both mice (24, 25) and humans (26, 27). Targeted genetic ablation of *Nampt* in adipose depots has established a role for NAD^+^ biosynthesis in thermogenic function (28) and adipose tissue plasticity in response to an obesogenic diet (29). *Nampt* also controls skeletal muscle function and integrity (30, 31), and different modalities of exercise training give rise to increased NAMPT abundance (32–34). Together, this indicates a role of NAMPT in both cellular metabolism and circadian biology. However, the effect of NAMPT on the clock has only been shown in vitro (22, 23), and in the liver (35). In this study, we describe the role of *Nampt* and NAD^+^ in vivo on the circadian control of adipose and skeletal muscle tissues. Unexpectedly, we find a complex tissue type–dependent regulation, in which NAMPT exerts substantially different circadian control between white and brown adipose tissues, while seemingly not affecting the circadian clock in skeletal muscle. We further explore the effects of adipose *Nampt* deficiency on circadian metabolism and tolerance to cold exposure.

## Results

### NAMPT-Dependent NAD^+^ Synthesis Regulates the Molecular Clock in a Tissue-Specific Manner.

**Fig. 1.**
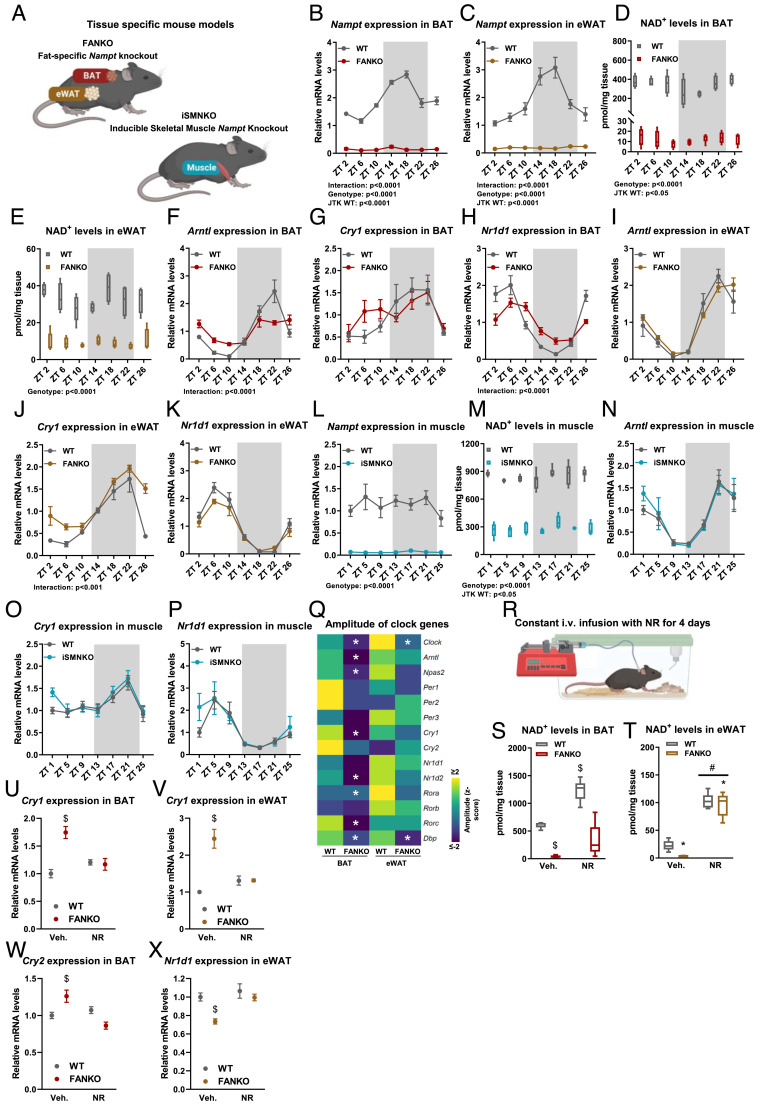
NAMPT-dependent NAD^+^ levels regulate the molecular clock in a tissue-specific manner. (*A*) Mouse models used to study the tissue-specific roles of *Nampt*-dependent NAD^+^ levels for the molecular clock: fat-specific *Nampt* knockout (FANKO) and inducible skeletal muscle-specific *Nampt* knockout mice (iSMNKO). Gene expression and NAD^+^ levels in tissues from 2 to 4-mo-old WT, and tissue-specific *Nampt* KO (FANKO, iSMNKO) male mice. Tissues were harvested at 4-h intervals over the course of 24 h, with ZT 0 denoting the start of the light phase. *Nampt* expression in (*B*) BAT and (*C*) eWAT (n = 4 to 5). NAD^+^ levels in (*D*) BAT and (*E*) eWAT (n = 4 to 5). *Arntl,* *Cry1* and *Nr1d1* expression in (*F*–*H*) BAT, and (*I*–*K*) eWAT (n = 4 to 5). (*L*) *Nampt* expression and (*M*) NAD^+^ levels in gastrocnemius muscle (n = 5 to 8). (*N*–*P*) *Arntl, Cry1* and *Nr1d1* expression in gastrocnemius (n = 5 to 8). Significant differences found by 2-way ANOVA or JTK rhythmicity are noted. Data from RNA sequencing performed on BAT and eWAT at ZT 6, 10, 18, and 22 (n = 4). (*Q*) Heatmap of amplitude of core clock genes, * denotes significantly different circadian rhythmicity between WT and FANKO. (*R*) Nicotinamide riboside (NR) intravenous infusion setup. NAD^+^ levels in (*S*) BAT and (*T*) eWAT after NR infusions in WT and FANKO mice (n = 5 to 6). *Cry1* expression in (*U*) BAT and (*V*) eWAT after NR infusions in WT and FANKO mice (n = 5 to 6). (*W*) *Cry2* expression in BAT after NR infusions in WT and FANKO mice (n = 5 to 6). (*X*) *Nr1d1* expression in eWAT after NR infusions in WT and FANKO mice (n = 5 to 6). Significant differences were found by 2-way ANOVA. * denotes significant difference between WT and FANKO. # denotes significant difference with treatment. $ denotes significant difference from WT vehicle treated.

To explore the role of NAMPT in regulating the clock in vivo, we used fat-specific *Nampt* knockout mice (FANKO) (29) and inducible skeletal muscle-specific *Nampt* knockout mice (iSMNKO) (Fig. 1*A* and *SI Appendix*, Fig. S1*A*). Both mouse lines exhibited similar bodyweights and body compositions (*SI Appendix*, Fig. S1*B*) (29), eating behaviors (*SI Appendix*, Fig. S1 *C* and *D*), and locomotor activities (*SI Appendix*, Fig. S1*E*) compared with littermate controls. Thus, FANKO and iSMNKO mice are ideal models to compare tissue-specific clock function in vivo. In BAT and epididymal WAT (eWAT) from wild-type (WT) mice, *Nampt* expression oscillated in a circadian manner and peaked during the dark phase (Fig. 1 *B* and *C*) consistent with earlier observations (36–38). This pattern of *Nampt* regulation resulted in significant NAD^+^ oscillation in BAT and a trend toward rhythmicity in eWAT (Fig. 1 *D* and *E*). In FANKO adipose depots, the markedly reduced NAD^+^ levels no longer exhibited any oscillatory pattern, supporting the foundational discovery in cell systems that *Nampt* is responsible for a circadian fluctuation in NAD^+^ levels (22, 23). However, even though NAD^+^ levels were reduced to comparable absolute levels in FANKO BAT and eWAT (12 and 9 pmol/mg tissue, respectively), the subsequent impact on the core clock machinery was different. In BAT, we observed pronounced dampening of both *Bmal1* (a.k.a. *Arntl*), and *Reverb-α* (a.k.a. *Nr1d1*) expression (Fig. 1 *F*–*H*). Conversely, *Arntl* and *Nr1d1* levels in FANKO eWAT were largely unaffected by *Nampt* deletion (Fig. 1 *I*–*K*). Clock genes in nonadipose tissues from FANKO mice were unchanged (*SI Appendix*, Fig. S1 *F*–*K*).

The tissue-specific nature of *Nampt* circadian regulation was even more evident when comparing the FANKO adipose clocks to the skeletal muscle clock in iSMNKO mice. Consistent with previous work (36, 38, 39), we observed no oscillation in skeletal muscle *Nampt* levels (Fig. 1*L*). Yet, even without *Nampt* oscillation, skeletal muscle NAD^+^ levels displayed a significant, albeit subtle, biorhythm (Fig. 1*M*), which may be due to diurnality of NAD^+^-consuming reactions. In iSMNKO muscle, *Nampt* was reduced to approximately the same level as in FANKO BAT and eWAT, and the relative reduction in NAD^+^ levels was 67% compared with 96% and 73% in FANKO BAT and eWAT, respectively. Like adipose tissues, NAD^+^ oscillation in skeletal muscle was totally abrogated by *Nampt* deletion (Fig. 1*M*). Nevertheless, the absolute level of NAD^+^ was still relatively high in iSMNKO muscles. Despite loss of *Nampt* and lower NAD^+^, the expression of *Arntl, Nr1d1*, and *Cry1* in iSMNKO skeletal muscle oscillated similarly compared with WT mice (Fig. 1 *N*–*P*). Thus, our findings reveal that the rhythmicity of *Nampt* and NAD^+^ and the NAMPT-dependence of core clock genes are differentially regulated across tissues.

To investigate the implications of *Nampt* ablation on a broader spectrum of circadian genes, we performed RNAseq analyses on BAT and eWAT harvested at four time points. We selected the midpoints (i.e., ZT 6 and ZT 18) in each phase as well as time points immediately preceding the transition between light/dark phases (i.e., ZT 10 and ZT 22). Notably, these transitional periods just prior to animals waking up and going to sleep have been shown to be transcriptional “rush hours” with the highest number of peaking circadian genes (36). Rhythmicity was analyzed using the R package LimoRhyde as it allows for assessments of oscillation and amplitude of nonsymmetrical data (40). *Nampt* ablation markedly diminished the amplitude of clock gene expression in FANKO BAT, particularly attenuating oscillation of *Clock*, *Arntl*, *Npas2*, *Cry1, Nr1d2, Rorc*, and *Dbp* (Fig. 1*Q*). Circadian amplitude was also blunted in FANKO eWAT and was most pronounced for *Clock* and *Dbp* expression (Fig. 1*Q*). In contrast to reduced amplitudes, the times at which clock genes peaked in both depots remained generally unaffected by *Nampt* deficiency (*SI Appendix*, Fig. S1*L*). Previously, knockout of *Nampt* in the liver was shown not to affect the rhythmicity of *Arntl, Clock and Per2* (35), whereas the amplitude of *Arntl* and *Per2* were decreased in U2-OS and NIH 3T3 cells in response to *SIRT1* knockdown (41). In another study, pharmacological inhibition of NAMPT by FK866 was shown to enhance the amplitude of *Per2* in mouse embryonic fibroblasts (23). The amplitude of *Clock* and *Arntl* was decreased in BAT, and *Per2* trended to be decreased (*SI Appendix*, Fig. S1*M*). In eWAT, only the amplitude of *Clock* was decreased (*SI Appendix*, Fig. S1*M*). In the skeletal muscle of iSMNKO mice, the rhythms of *Per2*, *Drp1*, and *Clock* expression were unchanged (*SI Appendix*, Fig. S2 *A*–*C*). This might be due to the relatively high level of NAD^+^ remaining in the skeletal muscle of iSMNKOs. To determine this, we measured core clock gene expression at ZT 4 in constitutive skeletal muscle *Nampt* knockout (SMNKO) mice and control littermates. In the skeletal muscle of SMNKO mice, NAD^+^ levels were depleted by 91% (30), but *Arntl*, *Nr1d1*, *Cry1*, *Per2*, *Dbp*, and *Clock* expression were still not impacted (*SI Appendix*, Fig. S2*D*). Together, our findings highlight the cell type–specific nature of NAMPT circadian influence and support that NAMPT acts to selectively potentiate the rhythmicity of the brown and white adipose molecular clocks in vivo.

### Nicotinamide Riboside Supplementation Partially Rescues the Core Clock.

To determine whether boosting NAD^+^ levels could restore clock gene expression in *Nampt*-depleted adipose tissue, mice were supplemented with the NAD^+^ precursor nicotinamide riboside (NR). Achieving constant high levels of NAD^+^ in the adipose tissues is challenging with oral or injection protocols, given that NAD^+^ has a high turnover rate in adipose tissues (42–44). Thus, we used a constant 4-d intravenous infusion of NR through a jugular catheter at a dose of 800 mg/kg/d (Fig. 1*R*) that led to a twofold and a fourfold increase in NAD^+^ levels in WT BAT and eWAT, respectively (Fig. 1*S* and *T*). To assess the capacity of NR supplementation to rescue clock function in our NAMPT KO model, adipose tissues were harvested at ZT 6, where we had previously observed a difference in clock gene expression between FANKO mice and control littermates (*SI Appendix*, Fig. S1*M*). NR rescued NAD^+^ levels in FANKO BAT substantially and in FANKO eWAT to even higher levels than those found basally in WT mice (Fig. 1 *S* and *T*). NR led to a complete rescue of *Cry1* in both BAT and eWAT of FANKO mice (Fig. 1 *U* and *V*), whereas restoration of clock genes, *Cry2* and *Nr1d1*, was depot specific (Fig. 1 *W* and *X* and *SI Appendix*, Fig. S2 *E* and *F*). Additionally, NR decreased expression of *Arntl* and *Npas2* expression in both genotypes and adipose depots (*SI Appendix*, Fig. S2 *G*–*J*), in stark contrast to the effect observed in response to *Nampt* deletion. Thus, acutely elevating NAD^+^ levels by constant infusion of NR partially restores long-term disruption of the circadian clock and underscores the tight regulatory connection between NAD^+^ levels and the core clock.

### NAMPT Is Essential for Global Adipose Transcriptional Rhythmicity.

**Fig. 2.**
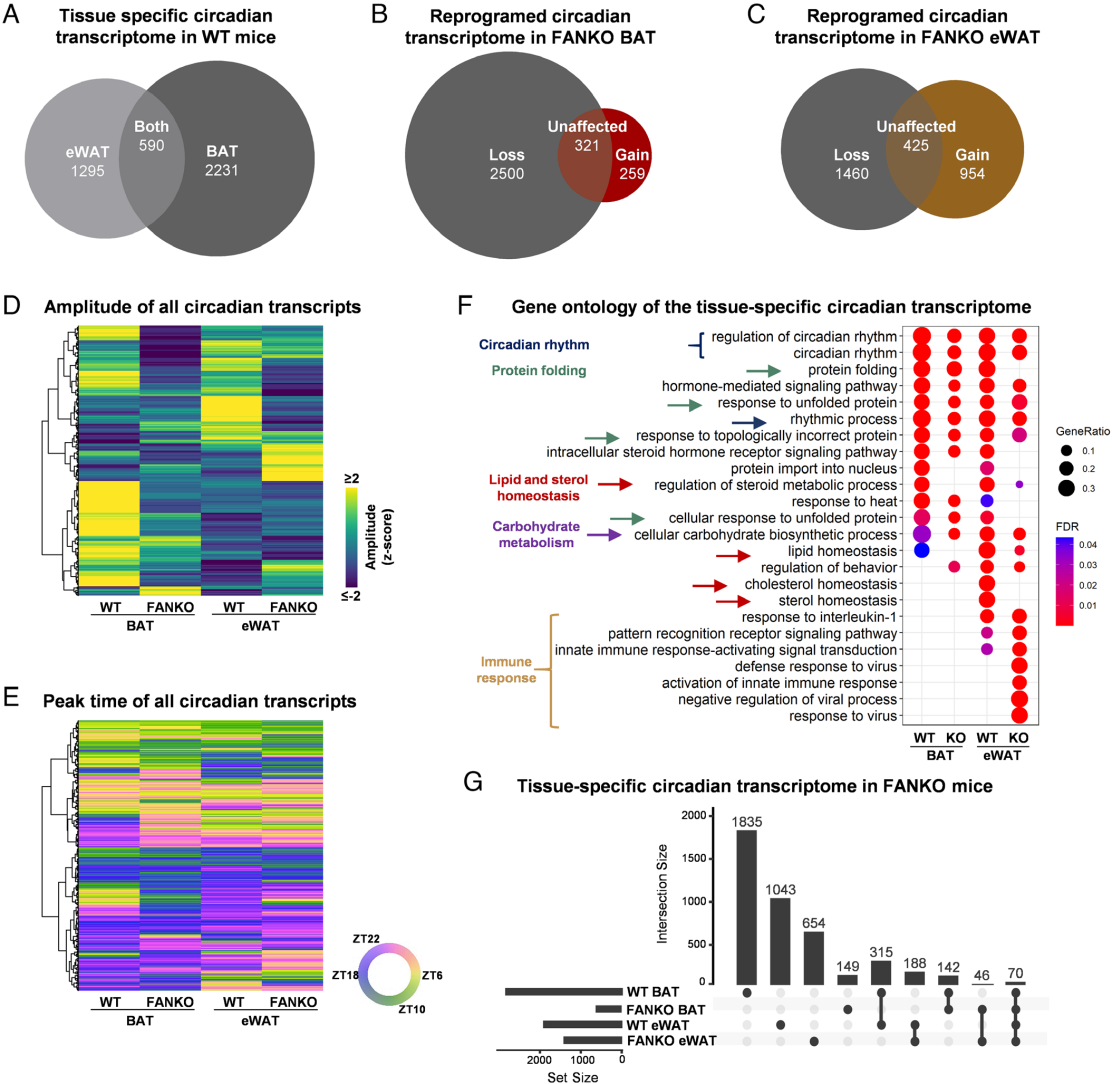
*Nampt* controls global adipose transcriptional rhythmicity through changes in circadian amplitude. Data from RNA sequencing performed on BAT and eWAT at ZT 6, 10, 18, and 22 from Fig. 1. (n = 4). (*A*) Euler diagram of oscillating transcripts in WT BAT and eWAT. (*B*) Euler diagram of oscillating transcripts in WT and FANKO BAT. (*C*) Euler diagram of oscillating transcripts in WT and FANKO eWAT. (*D*) Heatmap of amplitude and (*E*) peak time of rhythmic transcripts in BAT and eWAT of WT and FANKO mice. (*F*) Gene ontology analysis of circadian genes in all four groups. (*G*) UpSet plot showing selected overlaps of circadian rhythmicity of genes in the four groups. All analyses were performed with FDR cutoff < 0.01.

We next explored how NAMPT regulation of the core clock affected global transcriptional programs. Consistent with earlier studies (36), we found a higher number of genes to be circadian in BAT (2,821) than in eWAT (1,885) (Fig. 2*A* and Dataset S1), of which only 590 were shared between the depots (Fig. 2*A*). *Nampt* deletion substantially reduced the BAT circadian transcriptome by 2,500 genes (Fig. 2*B* and *SI Appendix*, Fig. S2*K*). FANKO eWAT lost rhythmicity of 1,460 genes relative to WT tissue (Fig. 2*C* and *SI Appendix*, Fig. S2*L*), indicating that NAMPT exerts marked control over general transcriptional rhythmicity in both brown and white adipose depots despite the difference in regulation of the core clock. In line with our findings on how NAMPT potentiates the oscillations of the core clock genes, the amplitudes of oscillating genes in BAT and eWAT were significantly reduced in FANKO mice (Fig. 2*D*) compared with WT littermates, whereas peak time were largely unchanged (Fig. 2*E*). Beyond its specific role in imparting transcriptional rhythmicity, *Nampt* deletion changed the overall expression level of 13,333 transcripts (6,801 down/6,532 up) in BAT and 10,063 transcripts (4,562 down/5,501 up) in eWAT. This impact on transcription is consistent with previous work by Yamaguchi and colleagues (28, 45) and highlights a broader requirement for NAD^+^ as an essential transcriptional regulator in addition to the molecular clock.

Gene ontology analysis of the circadian genes in the two adipose depots showed clear similarities. Terms connected to circadian rhythmicity and protein folding were highly enriched in both adipose tissue depots independent of genotype (Fig. 2*F*) but with a higher number of genes regulated in WT compared with FANKO mice. The unique intersection between the four datasets was visualized by an UpSet plot (Fig. 2*G*). A total of 315 genes, over half of the rhythmic genes common between the two adipose tissues (590 genes, Fig. 2*A*), lost rhythmicity in both depots in response to *Nampt* deletion (*SI Appendix*, Fig. S2*M*). The highest ranking ontology terms for these shared genes included biosynthesis of triglyceride, cholesterol and phosphatidic acid, lipid droplet organization, and cardiolipin metabolism (Dataset S2). These data underscore the importance of NAMPT in the regulation of circadian lipid metabolism in both tissues. As indicated in the UpSet plot, 46 genes gained rhythmicity in response to knockout of *Nampt* in both tissues. However, these appeared to be related to an immune response (Dataset S2).

There were also notable differences in the tissue-specific ontologies of circadian genes. In eWAT of the FANKO mice, genes involved in lipid and cholesterol homeostasis lost rhythmicity, whereas genes related to inflammation gained rhythmicity (Fig. 2*F* and Dataset S3). The increase in rhythmic inflammatory-related genes in the eWAT of FANKO mice may be due to infiltration of immune cells. In line with this, high-fat diet (HFD) substantially increased inflammation in WAT of the FANKO mice (29). The highest ranking terms between the genes gaining rhythmicity in response to *Nampt* deletion in BAT were immune response, and positive regulation of transcription by RNA polymerase II (Dataset S3). Looking at the ontology for genes losing rhythmicity in each of the two depots separately (Dataset S3), we found remarkable tissue-specific differences. In BAT, more than 100 genes related to each of the following terms lost rhythmicity: positive regulation of protein phosphorylation, positive regulation of transcription, cytoskeleton organization, positive regulation of cellular component organization, and organic acid metabolic process (Dataset S3). This result points to an effect of NAMPT in circadian regulation of general transcription and cytoskeleton organization in BAT. In eWAT, loss of rhythmicity was seen in a high number of genes related to cell cycle regulation and metabolism, explicitly the terms: regulation of cell cycle, organonitrogen compound metabolic process, cellular lipid metabolic process, phosphate-containing compound metabolic process, monocarboxylic acid metabolic process (Dataset S3). An analysis of the common rhythmic genes for the two adipose tissues revealed that more transcripts lost rhythmicity specifically in BAT compared with eWAT, 146 vs. 59, respectively (*SI Appendix*, Fig. S2*M*). Among the BAT-specific transcripts were again genes related to organic acid metabolic process, and positive regulation of transcription (*SI Appendix*, Table S2). These tissue-specific differences, due to *Nampt* deletion, in the number of oscillating transcripts and on their gene ontology highlight that the effect of NAD^+^ depletion on circadian rhythmicity largely depends on the cellular context, such as gene accessibility, or the presence of specific transcriptional regulators.

### *Nampt* Ablation Affects Adipose Metabolite Biorhythms in a Depot- and Diet-Specific Manner.

**Fig. 3.**
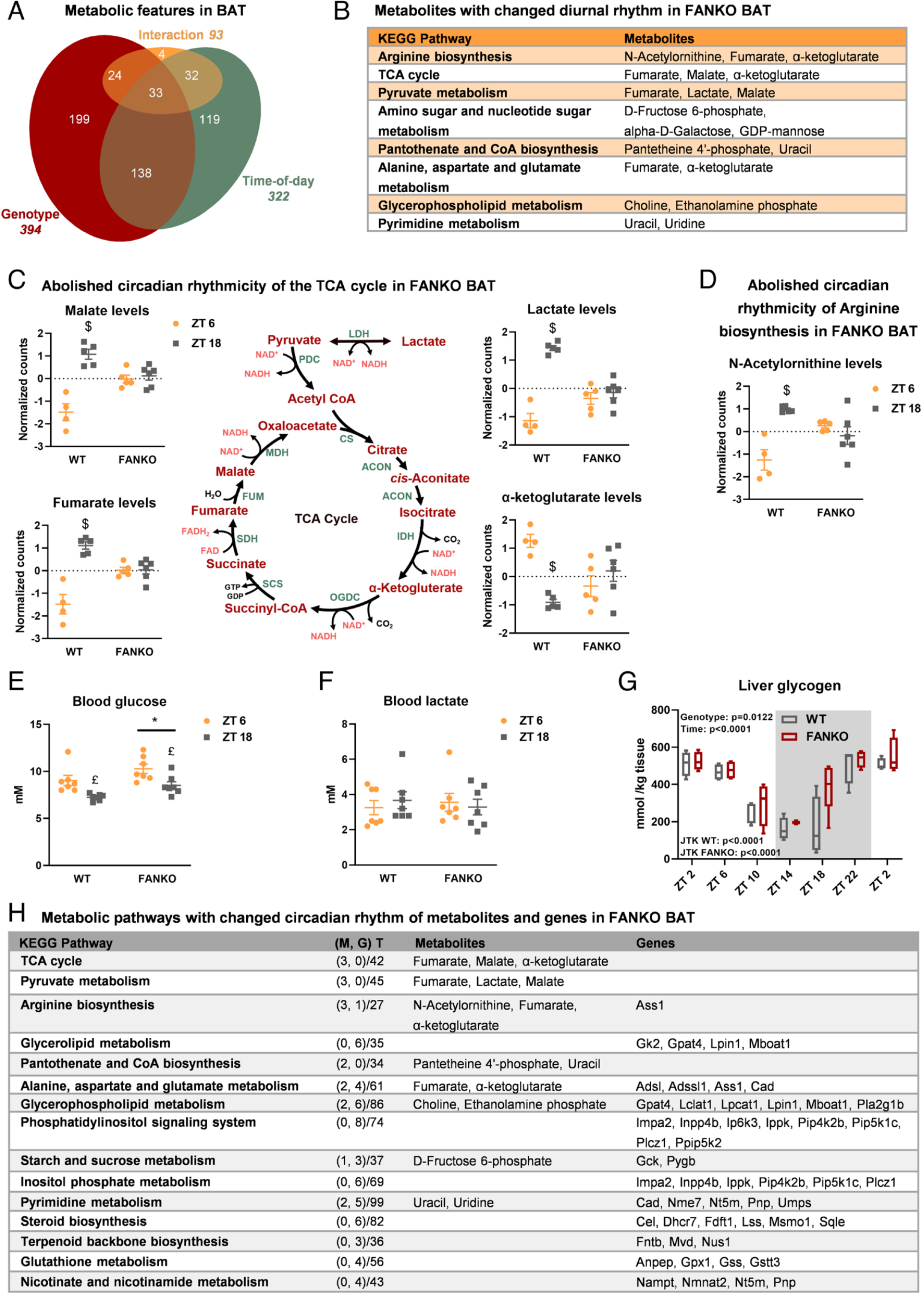
*Nampt* ablation affects metabolite rhythmicity in brown adipose tissue. Metabolomics analysis of BAT of WT and FANKO mice harvested at ZT 6 and ZT 18 (n = 4 to 6). (*A*) Euler diagram of metabolic features in BAT significantly affected by genotype, time-of-day, or an interaction between these two factors. (*B*) List of KEGG pathways with changed diurnal pattern in response to *Nampt* deletion in BAT. (*C*) Schematic overview of pyruvate metabolism and the TCA cycle, and plots displaying the content of metabolic intermediates with changed diurnal rhythm in BAT in response to *Nampt* deletion. (*D*) Levels of the arginine biosynthesis intermediate, N-acetylornithine, in WT and FANKO BAT. Measurements of (*E*) blood glucose and (*F*) blood lactate levels in WT and FANKO mice at ZT 6 and ZT 18 (n = 7). (*G*) Liver glycogen levels in WT and FANKO mice at 4-h intervals over the course of 24 h. (*H*) List of selected KEGG pathways regulated at the transcriptional and metabolic level. M: metabolite, G: gene, and T: total number of metabolites and genes in pathway. Significant differences found by 2-way ANOVA or JTK rhythmicity are noted. All metabolomics analyses were performed with a FDR cutoff < 0.05. * denotes significant difference between WT and FANKO. £ denotes significantly difference with time-of-day. $ denotes significant difference with time-of-day in WT.

Similar to circadian oscillations in gene expression, metabolite levels show a diurnal pattern in adipose tissue (46). The circadian rhythms of metabolite levels in BAT are profoundly affected by disruption of the molecular clock (46). Furthermore, depletion of NAD^+^ affects the metabolite profile in other model systems (31, 47, 48). To describe the effect of *Nampt* ablation on the diurnal metabolite rhythmicity of BAT, we performed metabolomics analyses on WT and FANKO mice tissues at the midpoint of the light and dark phases, ZT6 and ZT18, respectively. Any metabolite with concentration significantly changed between these two time points was characterized as rhythmic. Out of 760 metabolic features detected in BAT, 322 were rhythmic (Fig. 3*A*) and 171 of these oscillating metabolites were dependent on NAD^+^. *Nampt* deletion affected an additional 223 metabolites that were not classified as rhythmic. The critical importance of BAT NAD^+^ biosynthesis for metabolic rhythmicity was further underscored by principal component analysis (PCA). The WT metabolite profiles were clearly distinct at the two times of day, whereas loss of *Nampt* abolishes this distinction (*SI Appendix*, Fig. S3*A*, PC2). As many as 93 features showed an interaction between genotype and time of day (Fig. 3*A*). Among these specific features having different diurnal patterns in response to *Nampt* ablation were metabolites related to the KEGG pathways *Arginine biosynthesis*, *TCA cycle*, and *Pyruvate metabolism* (Fig. 3*B*). *Nampt* ablation completely abolished the diurnal rhythm of lactate and the three TCA cycle intermediates: α-ketoglutarate, malate, and fumarate (Fig. 3*C*). We observed similar patterns for N-acetylornithine, an intermediate in arginine biosynthesis and the urea cycle (Fig. 3*D*). These data indicate that NAMPT is a central regulator of circadian energy metabolism in BAT.

We next determined whether NAMPT-specific circadian changes in BAT metabolites were reflected in the blood. We measured blood glucose and lactate at the midpoint of each period (ZT6 and ZT18). Blood glucose was significantly higher in the FANKO mice at both times-of-day compared with controls, and both genotypes exhibited increased blood glucose during the day at ZT 6 compared with ZT 18 (Fig. 3*E*). Thus, the diurnal pattern of blood glucose levels was unaffected by *Nampt* deletion. Despite a clear effect in BAT, blood lactate levels did not differ between genotypes or time of day (Fig. 3*F*). To further investigate whether the rhythmicity of glucose metabolism in other tissues were affected by adipose *Nampt* deletion, we measured liver glycogen every 4 h. Liver glycogen levels were highly rhythmic in both genotypes, but the rhythmicity was unaffected by adipose *Nampt* deletion (Fig. 3*G*). Overall, these findings are consistent with earlier reports that adipose NAMPT plays a role in whole-body glucose metabolism (29, 49), yet the effect of *Nampt* deletion on metabolite rhythmicity appears to be more restricted locally within BAT.

Integration of the metabolomic analysis with the transcriptomic analysis allowed us to evaluate the overall impact of *Nampt* ablation on BAT circadian metabolism. Fig. 3*H* lists the most distinct KEGG pathways in BAT from the combined analysis of metabolites and genes with changed rhythmicity in response to *Nampt* ablation. The changes in TCA cycle and pyruvate metabolism were only seen on the metabolite level and were therefore likely driven directly by the decrease in NAD^+^ levels in the FANKO mice, given the role of NAD^+^ as a co-factor in these pathways (Fig. 3*C*). Three pathways, including *Aspartate metabolism, De novo pyrimidine biosynthesis*, and *Starch and sucrose metabolism,* were among the distinct KEGG pathways in the combined analysis that demonstrated a change in rhythmicity in response to *Nampt* ablation at both the metabolite and the transcriptional levels (Fig. 3*H*). In each case, the circadian expression of relevant enzymes were altered upstream of the changes in metabolite rhythmicity (*SI Appendix*, Fig. S3 *B*–*D*). The diurnal rhythms of several other metabolic pathways were only regulated at the transcriptional level. Among these were the *Phosphatidylinositol signaling system*, *Steroid biosynthesis*, and *Nicotinate and nicotinamide metabolism*. One reason for this discrepancy could be due to the higher resolution of the transcriptomic analysis. Alternatively, many genes cycle at the transcriptomic level, without having a diurnal pattern at the protein level. Concerning NAD^+^ metabolism, both nicotinamide and NAD^+^ levels were decreased in the FANKO mice, and the levels showed a time-of-day effect (*SI Appendix*, Fig. S3 *E* and *F*), although there was no difference in their diurnal patterns in response to *Nampt* ablation.

**Fig. 4.**
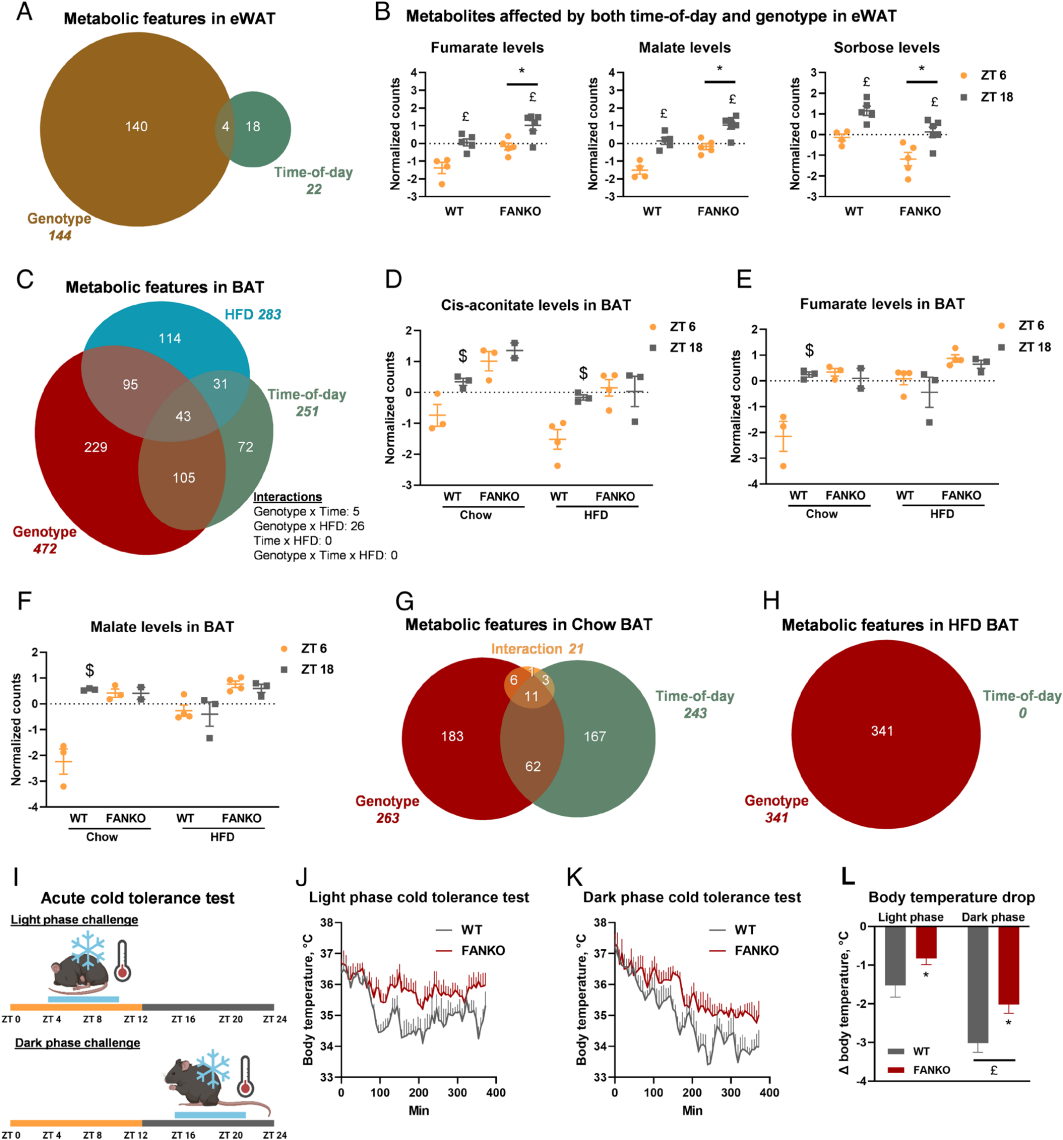
The effect of *Nampt* ablation on metabolite rhythmicity is tissue and diet dependent. Metabolomics analysis of eWAT from WT and FANKO mice harvested at ZT 6 and ZT 18 (n = 4 to 6). (*A*) Euler diagram of metabolic features in eWAT significantly affected by genotype, time-of-day, or an interaction between these two factors. (*B*) Metabolites affected by both genotype and time-of-day in eWAT. Metabolomics analysis of BAT of chow- and high-fat diet (HFD)-fed WT and FANKO mice harvested at ZT 6 and ZT 18 (n = 2 to 4). (*C*) Euler diagram of metabolic features significantly affected by genotype, time-of-day, and HFD as determined by three-way ANOVA. Day and night levels of (*D*) cis-aconitate, (*E*) fumarate, and (*F*) malate in BAT of WT and FANKO mice fed chow or HFD. (*G*) Euler diagram of metabolic features in BAT of chow-fed animals significantly affected by genotype, time-of-day, or an interaction between these two factors. (*H*) Euler diagram of metabolic features in BAT of HFD-fed animals significantly affected by genotype, time-of-day or an interaction between these two factors. (*I*) Schematic of acute cold tolerance test. (*J*) Light and (*K*) dark phase cold tolerance test. (*L*) Body temperature drop for the two cold tolerance tests. All metabolomics analyses were performed with FDR cutoff < 0.05. * denotes significantly difference between WT and FANKO. £ denotes significantly difference with time-of-day. $ denotes significant difference with time-of-day in WT at the same diet.

We next investigated NAD^+^-dependent metabolite oscillation on eWAT from the same WT and FANKO cohort used for BAT metabolomics. Of the 746 metabolic features detected in eWAT, 144 were altered by *Nampt* ablation and only 22 of these were rhythmic metabolites (Fig. 4*A*). This relatively nominal impact on eWAT metabolites was globally reflected in the PCA plot where there was no clear separation between genotypes or time-of-day (*SI Appendix*, Fig. S4*A*). While there was no interaction between the genotype and time-of-day effect in eWAT, fumarate, malate, and sorbose levels were significantly affected by both parameters (Fig. 4*B*). Collectively, these data illustrate the large difference in the diurnal pattern of metabolism between brown and white adipose depots, with 42% of the detected features cycling in BAT and only 3% cycling in eWAT. Moreover, our metabolomic findings are consistent with the transcriptional data emphasizing that NAMPT-dependent NAD^+^ biosynthesis plays a pronounced tissue-specific role in BAT rhythmicity.

NAMPT is essential for the function of adipose tissue (29, 49, 50), particularly apparent in the context of diet-induced obesity, where mice deficient in adipose *Nampt* fail to expand their adipose tissue to accommodate the lipid burden (29). Obesity is itself a major effector on the clock; in WT mice, high-fat diet (HFD) alters the clock in peripheral tissues including adipose and increases activity level and food intake of the animals during the light phase, thus attenuating the diurnal pattern (51–54). Furthermore, *Nampt* diurnal oscillation in adipose tissue is dampened in a genetic model of obesity (37), and the circadian metabolite profile of BAT is heavily influenced by HFD (55). Therefore, we sought to describe the effect of *Nampt* deletion on the diurnal metabolomic profile of BAT in the context of a HFD challenge. We performed metabolomic analysis on mice fed either chow or HFD and collected tissues at ZT 6 and ZT 18. We detected 1,028 metabolic features, 472 were affected by genotype, 251 by time-of-day, and 283 by diet (Fig. 4*C*). PCA revealed a clear separation between genotypes on both diets, but only mice fed a chow diet separated with time-of-day (*SI Appendix*, Fig. S4*B*). There were no significant FDR-corrected 3-way interactions, but 43 metabolite features were affected by the three factors: genotype, time-of-day, and diet, individually. Among these was the TCA intermediate cis-aconitate (Fig. 4*D*), which was rhythmic in WT BAT under both chow and HFD conditions but lost rhythmicity upon *Nampt* depletion. For two other TCA intermediates, fumarate and malate, loss of *Nampt* flattened rhythmicity on chow diet, mirroring the circadian-disrupting effects of HFD (Fig. 4 *E* and *F*). Comparing the genotype and time-of-day effects separately in the cohorts on chow and HFD gave a striking result. In the chow-fed cohort, 262 metabolic features were affected by genotype, 243 were affected by time-of-day, and 21 showed an interaction between genotype and time-of-day (Fig. 4*G*). This is a slightly lower number of regulated features compared with the earlier study (Fig. 3*A*). By contrast, in mice fed a HFD, 314 features exhibited a genotype effect, but no metabolic feature showed any significant diurnal rhythm (Fig. 4*H*). A strong effect of HFD on metabolite rhythmicity in BAT has been reported (55), but not to this extent. This emphasizes that on top of tissue specificity, diet adds another layer of complexity to the circadian regulation of metabolism.

### Thermogenic Capacity, but Not Rhythmicity, Is Affected by Adipose *Nampt* Deletion.

We next investigated the physiological consequences of the striking alterations in both circadian transcription and metabolism in *Nampt*-deficient adipose tissue. The main function of murine BAT, the tissue where we observed the larges changes in transcription and metabolism, is thermogenesis and body temperature defense (56). To investigate the effect of *Nampt* depletion on temperature regulation, we surgically implanted telemetric temperature probes in the intraperitoneal cavity of WT and FANKO mice. This enabled us to continuously monitor body temperature. The rhythmicity of body temperature was not changed in the FANKO mice at room temperature (*SI Appendix*, Fig. S4*C*). We then challenged mice with 6-h bouts of cold exposure either during the day (at ZT 4) or during the night (at ZT 16) (Fig. 4*I*). Activity and food intake during the cold exposure were similar between the two time points and genotypes (*SI Appendix*, Fig. S4 *D* and *E*). Both genotypes exhibited differential sensitivity to cold at ZT 4 compared with ZT 16 (Fig. 4 *J*–*L*). Yet surprisingly, under these conditions, the FANKO mice were significantly less sensitive to cold at both times-of-day compared with control littermates (Fig. 4 *J*–*L*). There was no interaction between genotype and time-of-day, indicating that the diurnal rhythmicity of cold sensitivity was independent of adipose NAMPT.

## Discussion

While it is widely accepted that NAMPT and the circadian clock are functionally linked, it has not been explored how ubiquitous the role is for NAMPT in the core clock across cell types. Here, we show that the regulation of the clock and metabolic biorhythms by NAMPT in vivo is highly tissue specific. Adipose *Nampt* ablation leads to dampening of several core clock genes in both brown and white adipose tissues as well as disruption of the circadian rhythmicity of a significant number of metabolic genes, especially those related to lipid metabolism. One such set of genes influences synthesis of the critical phospholipid, cardiolipin, which we have previously found to be sufficient to modulate adipocyte thermogenic capacity (57). In the present study, we find that circadian amplitude, but not phase or peak time, is affected by loss of *Nampt* in adipose depots. On the contrary, we find that the skeletal muscle clock is completely refractory to *Nampt* deletion.

Our results in adipose tissue are consistent with a recent study showing that *Nampt* knockout in the liver dampens circadian oscillations (35). The loss of rhythmicity in response to *Nampt* deletion was substantially higher in BAT (89%) and eWAT (77%) compared with the liver, where a loss of 26% of the rhythmic genes was observed (35). One of the likely mechanisms by which NAMPT-dependent NAD^+^ biosynthesis regulates the clock is through SIRT1-mediated deacetylation of PER2, which has been shown to modulate circadian amplitude (21). Other mechanisms by which NAMPT-dependent NAD^+^ biosynthesis can regulate circadian transcription are through SIRT1-mediated deacetylation of BMAL1 (20), PARP1-mediated ADP-ribosylation of CLOCK (58), and SIRT6-mediated recruitment of CLOCK:BMAL1 to certain circadian gene promoters (59). Conversely, elevating NAD^+^ levels, either by supplementation of NAD^+^ precursors (24, 35) or genetic deletion of the NAD^+^-dependent hydrolase CD38 (60), increases clock amplitudes. In agreement with this, we observed that constant infusion with NR partially rescued clock gene expression in BAT and fully rescued it in eWAT. However, while NR infusion was able to bring NAD^+^ levels in the *Nampt* KO tissues back to or above levels in control littermates, one consideration is that the continuous nature of the supplementation in this study would likely attenuate physiological NAD^+^ biorhythms and could, thus, potentially shift or impact transcriptional oscillations compared with WT mice. Therefore, increasing or boosting NAD^+^ levels in a pulsatile manner, aligning with physiological NAD^+^ biorhythms, would likely be the more viable supplementation strategy for augmenting adipocyte clock function under obesity and other contexts in which the clock is compromised.

Notably, exogenous NAD^+^ will not only feed into the clock and NAD^+^ feedback cycle. Beyond directly affecting the transcription of clock genes via enzymes, such as SIRT1/6 or PARP1, oscillating NAD^+^ will also impart circadian regulation through its role as a cofactor for other proteins and as a substrate to produce NADH and NADP(H) for electron transport, biosynthesis, and oxidative defense. This is likely reflected in the changes we observed in TCA intermediate rhythms, as rhythmicity was only observed at the metabolite level, including fumarate, malate, cis-aconitate, but not in gene expression of associated TCA enzymes. Several steps in the TCA cycle are NAD^+^ dependent, making NAD^+^ depletion a likely effector of the metabolic flow. Additionally, *Nampt* deletion could affect metabolic flux through posttranslational modification (PTM), such as SIRT3-mediated deacetylation of mitochondrial enzymes (61). Acetylation levels of all TCA enzymes are affected by *Bmal1* knockout, and acetylation of several of the enzymes follows a circadian pattern (62). NAMPT-dependent oscillation of TCA metabolites could also be mediated by regulation of anaplerotic or cataplerotic reactions linked to the TCA cycle. Several pathways feeding into or drawing from the TCA cycle, including *Arginine biosynthesis* and *Alanine, aspartate, and glutamate metabolism*, were regulated both at the transcriptional and metabolite level in FANKO brown adipose tissue (*SI Appendix*, Fig. S3*B*). This could also provide a means through which the diurnal changes in metabolism in response to *Nampt* deletion can feed back and regulate circadian transcription as has been observed previously (63, 64). However, the key considerations when integrating gene expression and metabolite levels are that a significant amount of differential circadian control occurs at the protein and PTM level (65). Therefore, future efforts will be needed to bridge the mechanistic underpinnings linking NAMPT-dependent circadian transcription to metabolite oscillation. Interestingly, we found that ablation of NAMPT-mediated NAD^+^ synthesis in mice on chow diet flattened metabolite biorhythms in BAT similarly to HFD, a nutritional challenge known to dramatically impinge on circadian physiology (66). Taken together, our data suggest that loss of NAD^+^ likely bidirectionally impacts transcriptional and metabolite rhythms at multiple levels, and future studies will be required to resolve specific contributions.

Despite the influence of NAMPT on circadian oscillation of transcripts and metabolites in BAT, we found that the diurnal rhythmicity of cold sensitivity was similar between FANKO mice and littermate controls. Surprisingly, loss of adipose tissue *Nampt* improved cold tolerance. Our findings differ from the increased cold sensitivity reported earlier using another floxed *Nampt* model (28). It was shown, using BAT-specific and pan-adipose *Nampt* KO mice, that WAT lipolysis, and not BAT metabolism, was the primary driver of cold intolerance (28). The likely reason for the different outcomes between our studies is experimental design. We exposed the animals to cold under fed conditions with ad libitum access to chow diet during the entire cold challenge, whereas the earlier study observed significant effects when challenging the adipose *Nampt* KOs under fasted conditions, a context that creates a stronger reliance on WAT lipolysis. Both studies reveal a significant influence of NAMPT-mediated NAD^+^ synthesis in adipose tissue on body temperature defense. Our findings indicate that, under fed conditions, loss of *Nampt* may be “overcompensated” and animals can defend body temperature even better than WT littermates. This compensatory increased cold tolerance does not appear to be driven by canonical thermogenic drivers, such as uncoupling in BAT (i.e., *Ucp1*) or in skeletal muscle (i.e., *Ucp2* or *Ucp3*) (*SI Appendix*, Fig. S4*F*). Nor does it appear to be caused by BAT gene programs linked to mitochondrial morphology (e.g., *Mfn1, Opa1, Fis1, Tfam)* and TCA activity (e.g., *Cs, Acon2, Idh2, Ogdh, Sucla2, Suclg1, Sdha, Sdhb, Sdhc, Sdhd, Fh1, Mdh1, Mdh2*) (*SI Appendix*, Fig. S4*G*). These genes are all down-regulated in *Nampt*-depleted BAT, in agreement with the observation by Yamaguchi et al (28). However, genes linked to the creatine futile cycle are increased in FANKO BAT (*SI Appendix*, Fig. S4*G*) and mRNA levels of *Ucp1* and the other mitochondrial related genes are increased in FANKO eWAT, all of which might contribute to the increased cold tolerance of mice lacking *Nampt* in adipose tissue. Notably, these potential compensatory measures are likely not circadian as the difference between FANKO and WT mice was observed during both the light and dark phases.

Given the seminal findings related to the effects of NAMPT on the clock in vitro (22, 23), the apparent lack of effect in the skeletal muscle clock is unexpected. Our observations indicate that oscillation in NAMPT-mediated NAD^+^ synthesis may not be required for regulation of all peripheral clocks. Notably, the NAD^+^ levels in iSMNKO muscle were not reduced to the same extent as seen in the constitutive adipose knockout models. However, the reduction in NAD^+^ levels in skeletal muscle in response to *Nampt* deletion was several folds larger than the diurnal difference in NAD^+^ levels. This suggests that the circadian oscillations in NAD^+^ levels do not affect clock gene expression in skeletal muscle. The data from the constitutive muscle knockout model further highlight that very low levels of NAD^+^ do not affect the muscle clock. The difference between skeletal muscle and adipose clocks in our study, therefore, likely reflects factors beyond the inducible or constitutive nature of *Nampt* deletion. *Nampt* oscillates in several adipose depots in both mice and baboons, whereas this oscillation is absent in skeletal muscle from both species (36, 38, 39). Therefore, the feedback cycle established between NAMPT/NAD^+^ and SIRT1/CLOCK:BMAL1 in vitro may not be a rhythmic driver of the transcription of circadian machinery in skeletal muscle. One of the key outstanding questions from our study is how tissue-specificity of NAMPT circadian control is orchestrated given that NAMPT itself is ubiquitously expressed. There are likely numerous direct and indirect underlying NAD^+^-dependent mechanisms that influence circadian transcriptional rhythmicity in different cell types (65), including but not limited to tissue-specific expression or cellular translocation (35) of downstream enzymes and regulatory factors that alter NAMPT-dependent circadian trajectory by changing protein levels or PTM. Other potential mechanisms might include tissue-specific NAD^+^ redox balances and thus different downstream circadian output (67). Thorough proteomic and PTM mapping will be required to uncover individual components that confer in vivo tissue specificity.

It is tempting to speculate that this tissue-specific dependence of NAMPT in the clock may have an evolutionary foundation. Skeletal muscle is a critical organ for mediating fight-or-flight response and must be ready to perform quickly at any time of the day to ensure survival. The activity of BAT, on the other hand, follows a strong diurnal pattern with increased glucose and lipid uptake in the end of the inactive phase (68, 69). The increase in BAT activity just before the active phase has been postulated as a means of initiating increases in body temperature and arousal prior to waking in mice (70) and humans (71).

From a broader perspective, our study highlights the necessity of testing cellular clock findings in different organs in vivo to determine their ubiquitous versus tissue-specific roles in circadian biology. This is further evidenced by the unique metabolic biorhythms induced across tissues following nutrient stress (72) or exercise at different times of day (73). Given recent work on NAMPT rhythmicity in the liver (35) and heart (74), future studies will be needed to fully resolve in which cell types NAMPT imparts circadian influence and to what extent.

## Materials and Methods

### Animals.

The tissue-specific *Nampt*-deficient mouse models were based on the Cre-lox system with exon 3 of *Nampt* (*Nampt*^tm1Jtree^) being flanked by loxP sites (75). To generate the fat-specific *Nampt* knockout (FANKO), the *Nampt*^tm1Jtree^ line was crossed with the Adiponectin-Cre mice (76) (010803, Jackson Labs), kindly provided by Prof. Karsten Kristiansen, as previously described (29). Inducible skeletal muscle-specific *Nampt* knockout mice (iSMNKO) were generated by crossing the *Nampt*^tm1Jtree^ line with the HSA-MCM line, which expresses a Mer-Cre-Mer (mutated estrogen receptor) chimeric protein (77) (025750, Jackson Labs). iSMNKO mice received tamoxifen (2 mg orally in 100 μL corn oil for three consecutive days) 7 wk prior to tissue harvest to induce *Nampt* deletion in skeletal muscle. Cre^-^ littermates were used as controls. Only male mice were used in the experiments.

Mice were housed at room temperature in an enriched environment with ad libitum access to water and chow (1310, Altromin) or high-fat diet (D12492, Research Diets). Lights in the housing facility were switched on at 6am and off at 6pm.

All animal experiments were approved by the Danish Animal Experiments Inspectorate under license no. 2014-15-0201-00181, 2018-15-0201-01441 (FANKO), and 2015-15-0201-00792 (iSMNKO).

### NAD^+^ measurements.

The assay was performed as previously described (78). In short, tissue was lysed in 400 μL 0.6 M perchloric acid. The supernatant was diluted in 100 mM Na_2_HPO_4_ (final pH 8.0), and NAD^+^ was measured on the diluted sample. A reaction mix [2% ethanol, 90 U/mL alcohol dehydrogenase, 130 mU/mL diaphorase, 10 μM resazurin, 10 μM flavin mononucleotide, 10 mM nicotinamide in phosphate buffer (100 mM Na_2_HPO_4_), pH 8.0] was added, and continuous resorufin accumulation was measured for 15 to 30 min by fluorescence excitation at 544 nm and emission at 580 nm. Absolute quantification was achieved through a standard curve made by serial dilution of a standard with a known concentration.

### Gene Expression.

Total RNA was extracted from tissues using TRI reagent (T9424, Sigma-Aldrich) and isolated using the RNeasy Mini Kit (74106, Qiagen). Reverse transcription was performed with the High Capacity cDNA Reverse Transcription kit (4368814, Applied Biosystems). Gene expression was determined by real time quantitative PCR using SYBR green (PP00259, Primerdesign). Expression levels were normalized to ssDNA input measured by a Qubit fluorometer (Thermo Fisher Scientific). Primer sequences are included in *SI Appendix*, *Supplemental Materials and Methods*.

### RNA Sequencing.

RNA sequencing libraries were prepared as described (79). Reads were subjected to 38-bp paired-end sequencing on a NextSeq500 (Illumina) and aligned using STAR v. 2.5.3a (80), against the GRCm38.p5 primary assembly and GENCODE comprehensive gene annotations (81) version 15. Details on the bioinformatics analysis are included in *SI Appendix*, *Supplemental Materials and Methods*.

### Metabolomics.

Sample processing and profiling was performed as described in *SI Appendix*, *Supplemental Materials and Methods*.

### Measurement of Blood Parameters.

At ZT 6 and ZT 18 blood glucose and lactate levels were measured directly with Contour Next (Bayer) and Lactate Plus (Nova Biochemical) strips, respectively.

### Measurement of Hepatic Glycogen Levels.

Glycogen was extracted from mouse liver samples by acidic extraction using 1 M HCl at 95 °C for 2 h. After a quick spin, tissue extracts were neutralized using 1 M NaOH followed by centrifugation at 16,000 g for 20 min at 4 °C. The supernatant was transferred to new tubes and a reaction mix consisting of 200 mM Tris-HCl, 500 mM MgCl_2_, 5.2 mM ATP, 2.8 mM NADP, and 6 μg/mL hexokinase and glucose-6-phosphate dehydrogenase mixture (10737275001, Roche Diagnostics) were added. After 15 min of incubation at RT, the glucose generated from the hydrolyzed glycogen was measured spectrophotometrically at 340 nm (Hidex sense, Hidex). All samples were measured in duplicates along with glucose standards.

### Indirect Calorimetry.

Indirect calorimetry was performed using the Phenomaster Home Cage System (TSE Systems). Animals were acclimated in the system for more than 5 d prior to the measurement. All data were recorded in 6-min intervals. Data collection was integrated into the TSE software. Basal food intake is an average over five constitutive days.

### Telemetry.

Body temperature and gross motor activity was measured using G2 E-Mitters surgically implanted into the intraperitoneal cavity. Mice were anasthetized with 2% isoflurane during surgery and were allowed to recover for 2 wk before the measurements. Signals from G2 E-Mitters were detected by ER400 Energizer/Receivers (STARR Life Sciences Corp.), and data collection was integrated into the TSE software. Basal physical activity is an average over five constitutive days. For the acute cold tolerance test, mice were transferred from room temperature to new cages in the Phenomaster, which were cooled down to 4 °C. Cold challenges were performed both at ZT 4 and ZT 16, and the mice were kept in the Phenomaster at 4 °C for 6 h.

### Constant Infusions with NR.

To obtain continuous intravenous (IV) infusions, a catheter was inserted in the right external jugular vein. The catheter was externalized through a dorsal incision in the interscapular region using a single-channel Vascular Access Button (VABM1B/25; Instech Laboratories). Mice were anasthetized with 2% isoflurane during surgery and were allowed to recover for 1 wk before the beginning of the infusions. Based on a pilot experiment, a NR (Elysium Health) dose of 800 mg/kg bodyweight/d was chosen. The infusate had an NR concentration of 22.4 g/L, and a pH of 7.2. After 4 d of constant infusions with NR or saline in their local environment, the mice were taken down at ZT 6.

## Statistical Analyses.

Statistical analysis was performed in GraphPad Prism v. 9.3.1 by 2-way or 3-way ANOVA with Sidák's multiple comparisons testing as the post hoc test, for testing differences between night and day. Oscillation in NAD^+^ levels and gene expression detected by qPCR in Fig. 1 was assessed in R using JTK (82). For RNA sequencing data, FDR < 0.01 was considered statistically significant. The metabolomics data were assessed by 2-way or 3-way ANOVA in R, and an FDR < 0.05 was considered statistically significant. Data are presented as mean ± SEM. Unless otherwise noted, *P* < 0.05 was considered statistically significant.

## Supplementary Material

Appendix 01 (PDF)

Dataset S01 (XLSX)

Dataset S02 (XLSX)

Dataset S03 (XLSX)

## Data Availability

The RNA-Seq dataset is available at the Gene Expression Omnibus database (GSE221550) (83). Codes for R figure generation, and RNA-Seq and metabolomic analysis are available on https://github.com/CBMR-Single-Cell-Omics-Platform/NAMPT-mediated-NAD-biosynthesis-controls-circadian-metabolism-in-a-tissue-specific-manner. RNA levels of the core clock genes in skeletal muscle of 4-wk-old SMNKO and WT littermates (*SI Appendix*, Fig. S2*D*) are extracted from RNA sequencing data GSE147453 (84) published in ref. 30.
