# Supplementary material for: NAMPT-dependent NAD+ biosynthesis controls circadian metabolism in a tissue-specific manner
Source: Proc Natl Acad Sci U S A. 2023 Mar 30;120(14):e2220102120. doi: 10.1073/pnas.2220102120 (PMC10083581; doi:10.1073/pnas.2220102120)
Supplement: Supplementary file 1 — Appendix 01 (PDF) [file pnas.2220102120.sapp.pdf]

PNAS

Supporting Information for

NAMPT-dependent NAD$^+$ biosynthesis controls circadian metabolism in a tissue-specific manner

Astrid L. Basse, Karen N. Nielsen, Iuliia Karavaeva, Lars R. Ingerslev, Tao Ma, Jesper F. Havelund, Thomas S. Nielsen, Mikkel Frost, Julia Peics, Emilie Dalbram, Morten Dall, Juleen R. Zierath, Romain Barrès, Nils J. Færgeman, Jonas T. Treebak, Zachary Gerhart-Hines

Jonas T. Treebak and Zachary Gerhart-Hines
Email: jttreebak@sund.ku.dk or zpg@sund.ku.dk

This PDF file includes:

Supporting Information on Materials and methods
Figures S1 to S4
Legends for Datasets S1 to S3
SI References

Other supporting materials for this manuscript include the following:

Datasets S1 to S3

Supporting Information on Materials and methods

Gene expression, Primers

**Gene**	**Forward**	**Reverse**
Arntl	TAGGATGTGACCGAGGGAAG	TCAAACAAGCTCTGGCCAAT
Clock	CCCTTCCTCCACACCGACA	GGACGACCTCCGCTGTGT
Cry1	CCATCCGCTGCGTCTATATC	AAGCAAAAATCGCCACCTGT
Cry2	CCATCGTCAATCATGCAGAG	CCACACAGGAAGGGACAGAT
Drp1	ACCGGGATCAGCGCCTCCAT	CAAGCGGGCTGTGGGTCGAG
Nampt	TGCCGTGAAAAGAAGACAGA	ACTTCTTTGGCCTCCTGGAT
Npas2	AAGGATAGAGCAAAGAGAGCCT	CATTTTCCGAGTGTTACCAGGG
Nr1d1	GTCTCTCCGTTGGCATGTCT	CCAAGTTCATGGCGCTCT
Per2	CCAGCTGCTAATGTCCAGTG	ACAGCAAACATATCCGCGTT
Ucp2	GCGCCTTCTACAAGGGGTTC	CTCCCGAGATTGGTAGGCAG
Ucp3	TGACCTGCGCCCAGC	CCCAGGCGTATCATGGCT

Western blot analysis

Protein was extracted from mouse adipose tissues and skeletal muscle by steel bead homogenization (Tissue Lyser II, Qiagen) in an ice-cold lysis buffer for adipose tissue (RIPA lysis buffer (20-188, Millipore), 0.1% SDS) and skeletal muscle (pH 7.4, 10% glycerol, 1% IGEPAL, 50 mM Hepes, 150 mM NaCl, 10 mM NaF, 1 mM EDTA, 1 mM EGTA, 20 mM sodium pyrophosphate, 2 mM sodium orthovanadate, 5 mM nicotinamide, 4 µM Thiamet G, and protease inhibitors (SigmaFast)). Homogenates were incubated on ice for 30 min (adipose tissue) or run end-over-end for 45 min at 4°C (skeletal muscle), followed by centrifugation at 16,000 *g* at 4°C for 15 min. The supernatant was stored in aliquots at 80°C until further analysis. Protein concentration was measured by BCA (23223 and 23224, Thermo Scientific). Western blot analyses were performed as described (1). Protein abundance was detected by immunoblotting using antibody for NAMPT (A300–372A, Bethyl).

RNA sequencing

Read summation onto genes was performed by featureCounts v. 1.5.3 (2). Differential expression testing was performed with edgeR (3). The model used for differential expression testing was of the *form ~ 0 + group + group:(ZT_sin + ZT_cos)*, where *group* encoded both tissue and genotype, and the parameters *ZT_sin* and *ZT_cos* are sine and cosine terms describing time, as generated by LimoRhyde (4). Circadianicity was found by testing for significance of the sine and cosine components. Differential rhythmicity was found by testing for differences in the sine and cosine components. All tests used the quasi-likelihood tests in edgeR. Gene ontology enrichment tests were performed using the topGO package (5).

Heatmaps were generated using the pheatmap package (6), amplitudes were Z-score transformed prior to plotting. Overlaps between groups was visualized using upset plots from the UpSetR package (7) and visualization of gene ontologies was performed by the dotplot function from the enrichplot package (8). The remaining figures were generated by the ggplot2 package of R (9). Predicted expression patterns, time of peak and amplitudes were calculated based on the parameters fitted by edgeR.

Metabolomics

Metabolites from BAT and eWAT were extracted in methanol:acetonitrile:water (5:3:2, 250 µL per 25 mg of tissue). Samples were sonicated, thoroughly vortexed, and centrifuged (16,000 *g*, 20 min, 4°C). From this, 100 µL supernatant was transferred to new tubes before lyophilization. BAT and eWAT samples were resuspended in 20 µL and 35 µL of 1% formic acid, respectively, spun down to remove debris, and transferred to HPLC vails. An aliquot (5 µL) of each BAT and eWAT sample was transferred to a tissue-specific pool and used for quality control (QC).

Profiling of BAT and eWAT samples was conducted as described (10), with LC-MS and RP separation on an Agilent 1290 Infinity HPLC system (Agilent Technologies, Santa Clara, CA)

equipped with an Agilent Zorbax Eclipse Plus C18 column (2.1 x 150 mm, 1.8 mm) with a 50 mm guard-column.

Raw data was converted to mzML format using ProteoWizard (11) before imported into Mzmine (12) for data processing using ADAP chromatogram builder, Wavelets (ADAP) deconvolution, Deisotoping and Join alignment modules.

Features with < x5 average abundance in QC samples/blanks, not found in at least 80% of QC samples or in 50% of all samples were excluded. Remaining missing values were k-Nearest Neighbor imputed and QC signal normalized in MetaboAnalyst (13).

2-way and 3-way ANOVA was performed on raw metabolic features without further annotation. Features were annotated to Metabolomics Standard Initative level 3 (82) using MS/MS spectra databases from National Institute of Standards and Technology 17 (NIST17) and MassBank of North America (MoNA). Metabolic pathway analysis was performed in MetaboAnalyst (83) on annotated BAT metabolites from the features displaying a significant interaction between genotype and time-of day. A joint pathway analysis was performed in MetaboAnalyst on the same metabolites and transcripts from the RNA sequencing analysis showing a significant difference in rhythmicity in response to *Nampt* deletion in BAT.

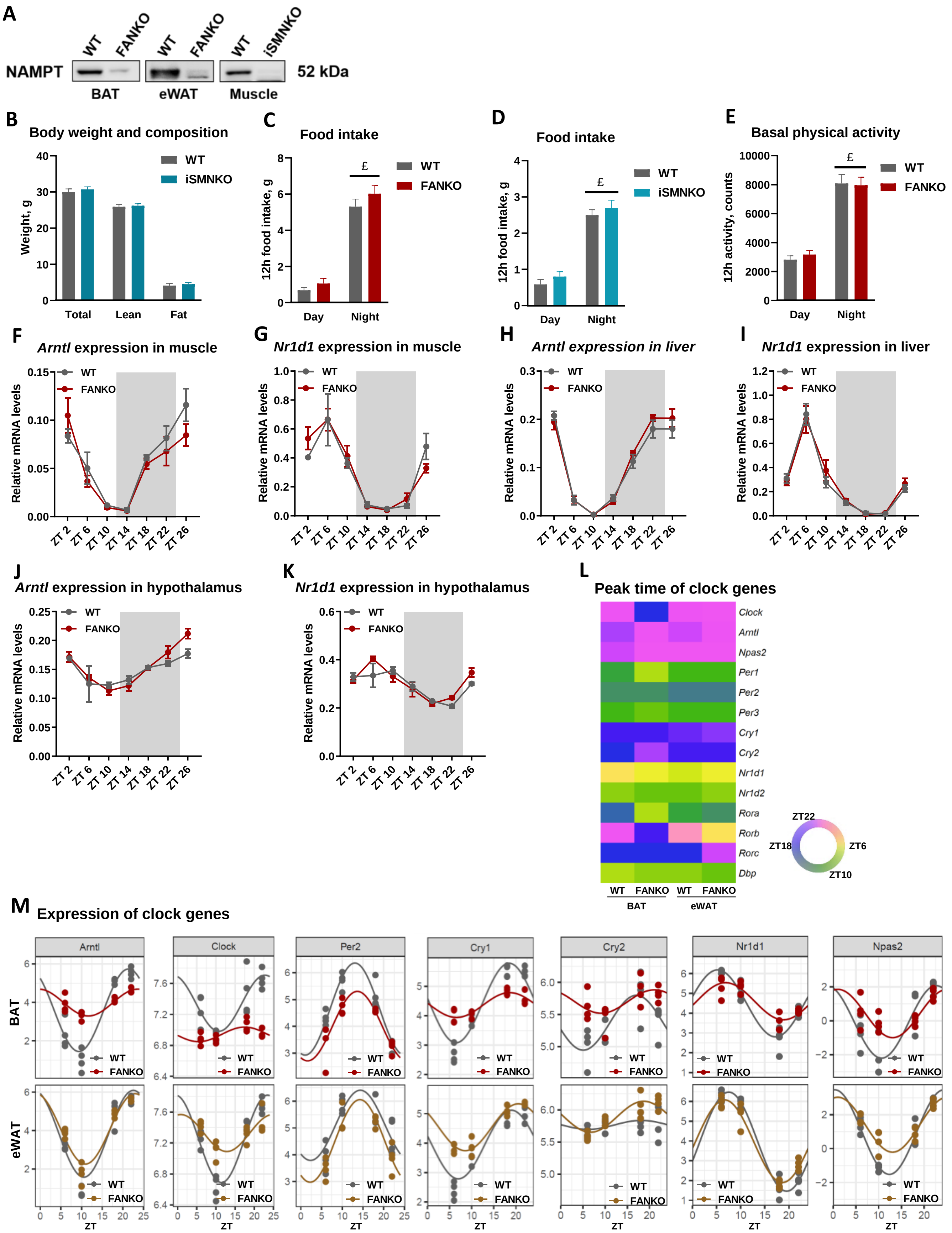
A
NAMPT
WT FANKO WT FANKO WT iSMNKO
52 kDa
BAT eWAT Muscle
B Body weight and composition
C Food intake
D Food intake
E Basal physical activity
F Arntl expression in muscle
G Nr1d1 expression in muscle
H Arntl expression in liver
I Nr1d1 expression in liver
J Arntl expression in hypothalamus
K Nr1d1 expression in hypothalamus
L Peak time of clock genes
Clock
Arntl
Npas2
Per1
Per2
Per3
Cry1
Cry2
Nr1d1
Nr1d2
Rora
Rorb
Rorc
Dbp
ZT22 ZT6 ZT10 ZT18
M Expression of clock genes
Arntl Clock Per2 Cry1 Cry2 Nr1d1 Npas2
BAT
eWAT
WT
FANKO
iSMNKO
ZT

Fig. S1. NAMPT-dependent NAD^+ levels regulate the molecular clock in a tissue-specific manner

Supplemental data to Fig. 1. (**A**) Protein levels of NAMPT in BAT and eWAT of WT and FANKO mice, and in gastrocnemius muscle of WT and iSMNKO mice. (**B**) Bodyweight and composition in WT and iSMNKO mice. Food intake in (**C**) FANKO and (**D**) iSMNKO mice. (**E**) Basal physical activity in WT and FANKO mice. Circadian expression of *Arntl* and *Nr1d1* in (**F, G**) soleus, (**H, I**) liver and (**J, K**) hypothalamus of WT and FANKO mice from Fig. 1. Data from RNA sequencing performed on BAT and eWAT at ZT 6, 10, 18 and 22 from Fig. 1. (n = 4). (**L**) Heatmap of peak time of core clock genes, with (**M**) graphical examples of *Arntl, Clock, Per2*, *Cry1*, *Cry2, Nr1d1,* and *Npas2* expression. £ denotes significant difference between night and day.

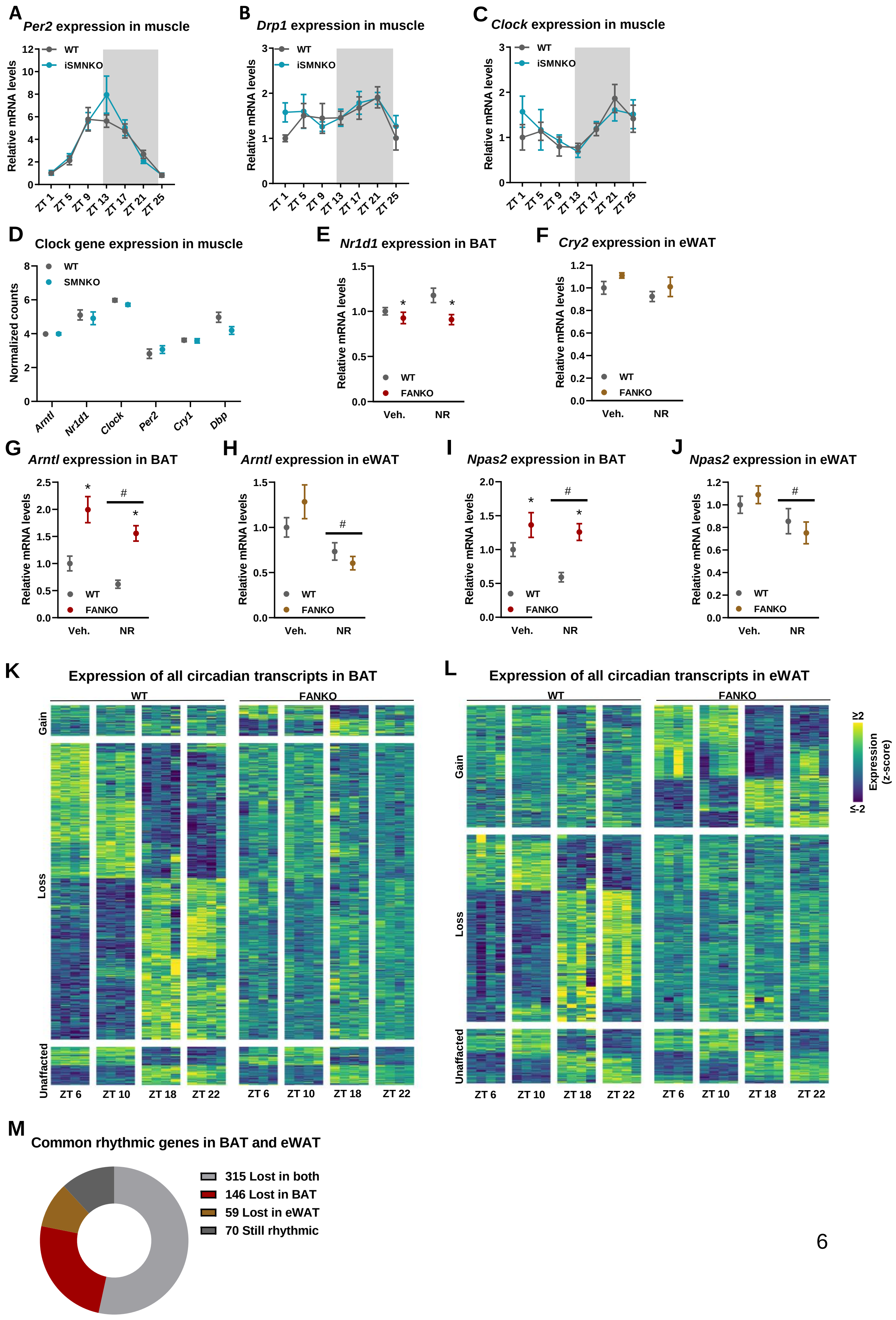

A
Per2 expression in muscle
B
Drp1 expression in muscle
C
Clock expression in muscle
WT
iSMNKO
Relative mRNA levels
ZT 1
ZT 5
ZT 9
ZT 13
ZT 17
ZT 21
ZT 25
D
Clock gene expression in muscle
WT
SMNKO
Normalized counts
Arntl
Nr1d1
Clock
Per2
Cry1
Dbp
E
Nr1d1 expression in BAT
F
Cry2 expression in eWAT
G
Arntl expression in BAT
H
Arntl expression in eWAT
I
Npas2 expression in BAT
J
Npas2 expression in eWAT
WT
FANKO
Veh.
NR
K
Expression of all circadian transcripts in BAT
L
Expression of all circadian transcripts in eWAT
WT
FANKO
Gain
Loss
Unaffacted
ZT 6
ZT 10
ZT 18
ZT 22
≥2
≤-2
Expression (z-score)
M
Common rhythmic genes in BAT and eWAT
315 Lost in both
146 Lost in BAT
59 Lost in eWAT
70 Still rhythmic

Fig. S2. *Nampt* controls adipose transcriptional rhythmicity through changes in circadian amplitude

Supplemental data to Fig. 1 and 2. Circadian expression of (**A**) *Per2*, (**B**) *Drp1*, and (**C**) *Clock* in gastrocnemius muscle of WT and iSMNKO mice from Fig. 1. (**D**) Expression of *Arntl*, *Nr1d1, Clock, Per2*, *Cry1*, and *Dbp* at ZT 4 in gastrocnemius muscle of WT and SMNKO mice. (**E**) *Nr1d1* expression in BAT after NR infusions in WT and FANKO mice (n = 5-6). (**F**) *Cry2* expression in eWAT after NR infusions (n = 5-6). *Arntl* expression in (**G**) BAT and (**H**) eWAT after NR infusions (n = 5-6). *Npas2* expression in (**I**) BAT and (**J**) eWAT after NR infusions (n = 5-6). Significant differences were found by 2-way ANOVA. * denotes significant difference between WT and FANKO. # denotes significant difference with treatment. Heatmap of all rhythmic transcripts in (**K**) BAT and (**L**) eWAT of WT and FANKO mice. (**M**) Fate of common rhythmic genes in BAT and eWAT in response to *Nampt*-depletion.

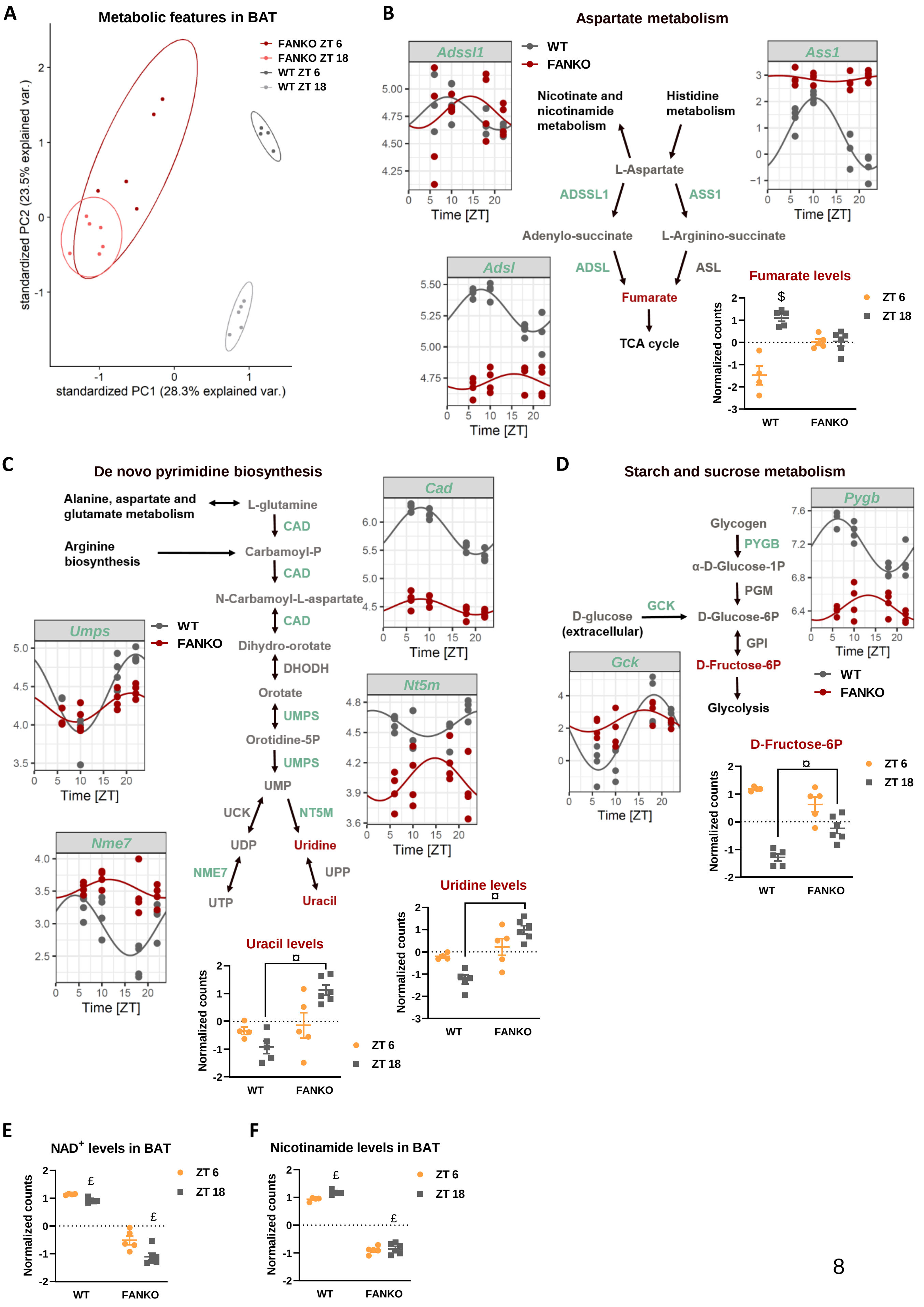
A
Metabolic features in BAT
FANKO ZT 6
FANKO ZT 18
WT ZT 6
WT ZT 18
standardized PC2 (23.5% explained var.)
standardized PC1 (28.3% explained var.)
B
Aspartate metabolism
Adssl1
Ass1
Adsl
WT
FANKO
Time [ZT]
Nicotinate and nicotinamide metabolism
Histidine metabolism
L-Aspartate
ADSSL1
ASS1
Adenylo-succinate
L-Arginino-succinate
ADSL
ASL
Fumarate
TCA cycle
Fumarate levels
$
ZT 6
ZT 18
Normalized counts
WT
FANKO
C
De novo pyrimidine biosynthesis
Alanine, aspartate and glutamate metabolism
L-glutamine
CAD
Arginine biosynthesis
Carbamoyl-P
CAD
N-Carbamoyl-L-aspartate
CAD
Dihydro-orotate
DHODH
Orotate
UMPS
Orotidine-5P
UMPS
UMP
UCK
NT5M
UDP
Uridine
NME7
UPP
UTP
Uracil
Cad
Umps
Nt5m
Nme7
WT
FANKO
Time [ZT]
Uracil levels
Uridine levels
¤
ZT 6
ZT 18
Normalized counts
WT
FANKO
D
Starch and sucrose metabolism
Glycogen
PYGB
α-D-Glucose-1P
PGM
D-glucose (extracellular)
GCK
D-Glucose-6P
GPI
D-Fructose-6P
Glycolysis
Pygb
Gck
WT
FANKO
Time [ZT]
D-Fructose-6P
¤
ZT 6
ZT 18
Normalized counts
WT
FANKO
E
NAD+ levels in BAT
£
ZT 6
ZT 18
Normalized counts
WT
FANKO
F
Nicotinamide levels in BAT
£
ZT 6
ZT 18
Normalized counts
WT
FANKO

Fig. S3. *Nampt* ablation affects metabolite rhythmicity in brown adipose tissue
Supplemental data to Fig. 3. (**A**) Principal component analysis (PCA) of metabolomics data from BAT of WT and FANKO mice harvested at ZT 6 and ZT 18 (n = 4-6). Schematic overview of (**B**) aspartate metabolism, (**C**) de novo pyrimidine biosynthesis, and (**D**) starch and sucrose metabolism, and plots displaying the level of enzymatic transcripts (green) and metabolites (red) with significantly changed diurnal rhythm in BAT in response to *Nampt* deletion. Day and night levels of (**E**) NAD^+, and (**F**) nicotinamide in BAT of WT and FANKO mice from the metabolomic analysis. $ denotes significant difference with time-of-day in WT. ¤ denotes significant difference between WT and FANKO at the same time-of-day. £ denotes a main effect of time-of-day.

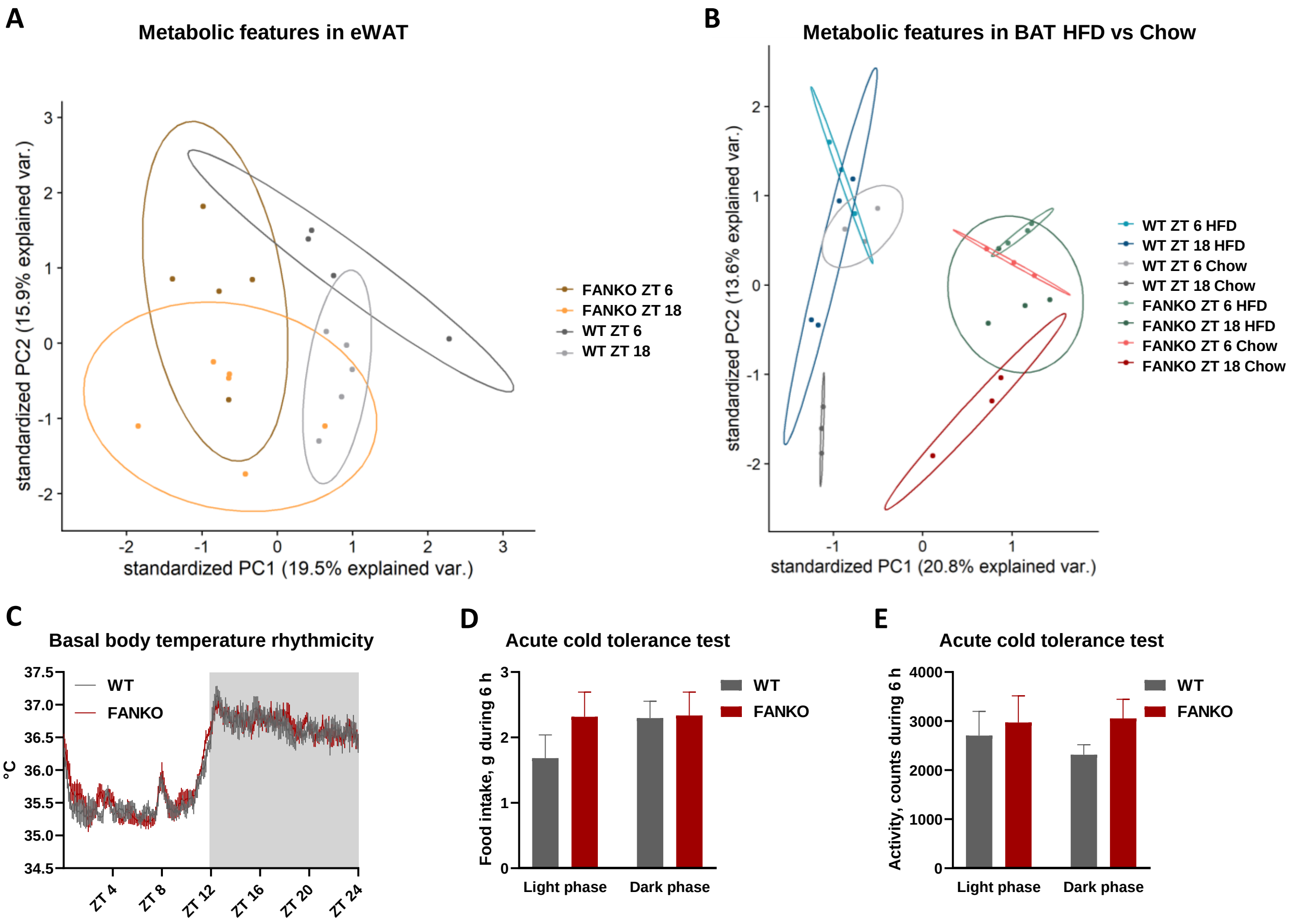

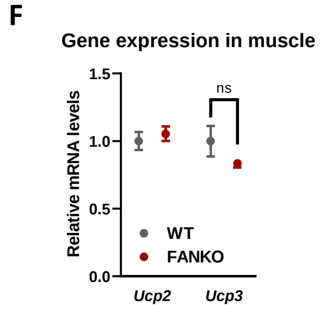

G

Changes in gene expression related to heat production in FANKO BAT and eWAT

Pathway	Genes	BAT	eWAT
**Energy expenditure**	*Ucp1*	↓ (FDR<0.01)	↑ (FDR=0.018)
**Mitochondrial morphology**	*Mfn1, Opa1, Fis1, Tfam*	↓ (FDR<0.01)	↑ (FDR<0.01)
**TCA cycle**	*Cs, Acon2, Idh2, Ogdh, Sucla2, Suclg1, Sdha, Sdhb, Sdhc, Sdhd, Fh1, Mdh1, Mdh2*	↓ (FDR<0.01)	↑ (FDR<0.01)
**Creatine futile cycle**	*Ckmt1, Ckb*	↑ (FDR<0.01)	*Ckmt1* ↓ (FDR<0.01)

Fig. S4. The effect of *Nampt* ablation on metabolite rhythmicity is tissue- and diet-dependent Supplemental data to Fig. 4. (**A**) PCA of metabolomics data from eWAT of WT and FANKO mice harvested at ZT 6 and ZT 18 (n = 4-6). (**B**) PCA of metabolomics data from BAT of chow- and HFD-fed WT and FANKO mice harvested at ZT 6 and ZT 18 (n = 2-4). (**C**) Basal body temperature rhythmicity in WT and FANKO mice. (**D**) Food intake and (**E**) basal activity during the two acute cold tolerance tests. (**F**) Expression of *Ucp2* and *Ucp3* at ZT 6 in gastrocnemius muscle of WT and FANKO mice. (**G**) Overview of changes in expression level of genes related to heat production in FANKO BAT and eWAT.

Dataset S1: Circadian transcripts in BAT and eWAT of WT and FANKO mice.

Dataset S2: Gene ontology enrichment analysis of the 590 common rhythmic genes in BAT and eWAT in Supplemental Fig. 2M. The genes are divided dependent on their response to *Nampt* deletion. Gene ontology terms mentioned in the Result section are highlighted with yellow.

Dataset S3: Gene ontology enrichment analysis of the rhythmic genes in BAT and eWAT of WT and FANKO mice. The four groups correspond to the two circles in Fig. 2B and the two circles in Fig. 2C. Gene ontology terms mentioned in the Result section are highlighted with yellow.

SI References

1. A. L. Basse, *et al.*, Skeletal Muscle Insulin Sensitivity Show Circadian Rhythmicity Which Is Independent of Exercise Training Status. *Frontiers in Physiology* **9** (2018).

2. Y. Liao, G. K. Smyth, W. Shi, featureCounts: an efficient general purpose program for assigning sequence reads to genomic features. *Bioinformatics* **30**, 923–930 (2014).

3. M. D. Robinson, D. J. McCarthy, G. K. Smyth, edgeR: a Bioconductor package for differential expression analysis of digital gene expression data. *Bioinformatics* **26**, 139–140 (2010).

4. J. M. Singer, J. J. Hughey, LimoRhyde: A Flexible Approach for Differential Analysis of Rhythmic Transcriptome Data. *Journal of Biological Rhythms*, 074873041881378 (2018).

5. A. Alexa, J. Rahnenführer, T. Lengauer, Improved scoring of functional groups from gene expression data by decorrelating GO graph structure. *Bioinformatics* **22**, 1600–1607 (2006).

6. R. Kolde, Pheatmap: pretty heatmaps. *R package version* **1**, 747 (2012).

7. J. R. Conway, A. Lex, N. Gehlenborg, UpSetR: An R package for the visualization of intersecting sets and their properties. *Bioinformatics* **33**, 2938–2940 (2017).

8. G. Yu, Enrichplot: visualization of functional enrichment result. *R package version* **1** (2019).

9. Hadley. Wickham, *Ggplot2 : elegant graphics for data analysis* (Springer, 2009).

10. E. G. Sustarsic, *et al.*, Cardiolipin Synthesis in Brown and Beige Fat Mitochondria Is Essential for Systemic Energy Homeostasis. *Cell Metabolism* **28**, 159-174.e11 (2018).

11. M. C. Chambers, *et al.*, A Cross-platform Toolkit for Mass Spectrometry and Proteomics. *Nat Biotechnol* **30**, 918–920 (2012).

12. T. Pluskal, S. Castillo, A. Villar-Briones, M. Oresic, MZmine 2: modular framework for processing, visualizing, and analyzing mass spectrometry-based molecular profile data. *BMC Bioinformatics* **11**, 395 (2010).

13. J. Chong, J. Xia, MetaboAnalystR: an R package for flexible and reproducible analysis of metabolomics data. *Bioinformatics* **34**, 4313–4314 (2018).
